# Evaluation de la qualité des concentrés de globules rouges produits au Centre National de Transfusion Sanguine de Lomé

**DOI:** 10.11604/pamj.2019.32.171.17680

**Published:** 2019-04-09

**Authors:** Hèzouwè Magnang, Essohana Padaro, Koffi Mawussi, Tama Ouenangou, Yao Layibo, Messanh Délagnon Irenée Kuéviakoé, Ahoefa Vovor

**Affiliations:** 1 Département des Sciences Fondamentales et Biologiques de la Faculté des Sciences de Santé de l’Université de Lomé (UL), Lomé, Togo; 2 Service des Laboratoires du Centre Hospitalier Universitaire Campus de Lomé, Lomé, Togo; 3 Service des Laboratoires du Centre Hospitalier Universitaire de Kara, Kara, Togo; 4 Centre National de Transfusion Sanguine de Lomé, Lomé, Togo; 5 Institut National d’Hygiène à Lomé, Lomé, Togo; 6 Service des Laboratoires du Centre Hospitalier Universitaire Sylvanus Olympio, Lomé, Togo

**Keywords:** Qualité, concentré de globules rouges, CNTS, Lomé, quality, concentrate of red blood cells, NCBT, Lomé

## Abstract

**Introduction:**

au Centre National de Transfusion Sanguine de Lomé, le sang total est systématiquement séparé en différents produits sanguins labiles. L'objectif de cette étude était d'évaluer la qualité des concentrés de globules rouges (CGR) qui y sont produits.

**Méthodes:**

il s'est agi d'une étude transversale réalisée de janvier à mars 2018 qui a porté sur 260 CGR (204 unités adultes et 56 unités pédiatriques). Les poches incluses ont été pesées afin de déterminer le volume de leur contenu. Le taux d'hémoglobine et l'hématocrite ont été déterminés en utilisant l'automate Pentra XLR de Horiba Médical. Nous avons évalué la fidélité et la justesse de l'automate afin d'assurer la fiabilité des mesures des variables analysées. Les analyses statistiques ont été réalisées à l'aide du logiciel R.

**Résultats:**

concernant les unités adultes, 79,90%; 81,86% et 43,13% des poches étaient conformes respectivement pour le volume, la teneur en hémoglobine et l'hématocrite. Au niveau des unités pédiatriques, 98,21%; 69,64% et 37,50% étaient conformes respectivement pour le volume, la teneur en hémoglobine et l'hématocrite. En considérant les trois paramètres simultanément, on avait un taux de conformité de 42,16% pour les CGR adultes contre 35,71% pour les CGR pédiatriques.

**Conclusion:**

nous recommandons d'élargir l'intervalle entre deux dons de sang et de mettre en œuvre le contrôle d'hémoglobine avant le don dans les critères d'aptitude au don de sang au CNTS de Lomé.

## Abstract (en)

**Introduction:**

at the National Center for Blood Transfusion (NCBT) in Lomé, whole blood is systematically separated into its various labile blood products. This study aims to assess the quality of the red blood cell concentrates (RBCC) produced.

**Methods:**

we conducted a cross-sectional study on 260 RBCCs (204 adult units and 56 paediatric units) from January to March 2018. The bags were weighed to determine the volume of their content. Hemoglobin and hematocrit levels were determined using the Horiba Medical Pentra XLR device. We evaluated the fidelity and precision of the device in order to ensure the accuracy of the measurements of the variables analyzed. Statistical analyses were performed using the R software.

**Results:**

adult units assessment showed that 79.90%; 81.86% and 43.13% bags were consistent with respect to the volume and haemoglobin and haematocrit levels. Paediatric units assessment showed that 98.21%; 69.64% and 37.50% bags were consistent with respect to the volume and haemoglobin and haematocrit levels. Simultaneous analysis of the three parameters showed a compliance rate of 42.16% for RBCCs in adults against 35.71% for pediatric RBCCs.

**Conclusion:**

we recommend to expand the interval between two blood donations and to perform hemoglobin test before blood donation in compliance with the eligibility criteria for giving blood at the National Center for Blood Transfusion in Lomé.

## Introduction

Dans la thérapeutique transfusionnelle, les concentrés de globules rouges (CGR) sont utilisés essentiellement dans les anémies symptomatiques avec pour but d'améliorer le taux d'hémoglobine (Hb) du patient [1, 2]. Pour assurer l'efficacité de la transfusion, il faut utiliser le bon produit chez le bon patient au bon endroit et au bon moment [3, 4]. Depuis l'année 2003 que le CNTS produit et distribue différents produits sanguins labiles, une seule étude de contrôle qualité de ces produits fut réalisée en 2006 [5]. Suite à cette étude, il a été recommandé de respecter les volumes de prélèvement chez les donneurs et inclure la mesure du taux d'hémoglobine dans la sélection médicale des donneurs de sang. Douze ans après l'étude de 2006, il y a eu une amélioration de la procédure de préparation des CGR mais le contrôle du taux d'hémoglobine pré don n'est toujours pas mis en place au CNTS de Lomé. Seul le volume de sang est respecté lors du prélèvement qui se fait à l'aide de balance pèse-poches, la sélection médicale des donneurs étant toujours basée sur l'état de coloration des conjonctives des candidats au don. Il était important de faire une nouvelle évaluation de la qualité hématologique des CGR produits au CNTS de Lomé. L'objectif général de ce travail était de déterminer la proportion de CGR produits au CNTS qui satisfait aux normes internationales de qualité.

## Méthodes

**Cadre d'étude:** ce travail a été réalisé dans deux établissements sanitaires. Le premier est le Centre National de Transfusion Sanguine de Lomé où ont été préparés les CGR qui ont été contrôlés. Le second est l'institut National d'Hygiène (INH) qui est l'un des laboratoires de référence au Togo. Tous les échantillons prélevés dans le cadre de cette étude ont été analysés à l'INH afin de déterminer les variables étudiées.

**Equipements et réactifs:** nous avons utilisé pour la détermination du volume des CGR la balance «Sartorius», modèle «TE6101». Le dosage des différents paramètres hématologiques a été fait grâce à l'analyseur d'hématologie Pentra XLR de Horiba médical. Cet appareil peut fonctionner soit en mode CBC (système de numération globulaire) soit en mode DIFF (CBC+formule leucocytaire) ou encore en mode RET (réticulocytes). Le taux d'hémoglobine et l'hématocrite de chaque CGR ont été déterminés à l'aide de cet automate en utilisant le mode CBC. Par ailleurs, nous avons utilisé: une strippeuse pour homogénéiser le sang de la poche et de tubulure avant de faire le prélèvement de l'échantillon de sang à analyser et une scelleuse pour fermer les tubulures après le prélèvement de l'échantillon de sang. Au niveau des réactifs, Pentra XLR est utilisé exclusivement avec les réactifs suivants: ABX diluent pour la dilution, le gainage et le nettoyage, ABX cleaner pour le nettoyage, ABX Lysebio pour la mesure de l´hémoglobine et ABX minoclair pour la procédure de nettoyage concentré.

**Type d'étude:** il s'agissait d'une étude transversale qui a duré trois mois (du 02 janvier au 31 mars 2018). La cible de cette étude était des concentrés de globules rouges préparés par le CNTS de Lomé.

**Critères de sélection:** étaient inclus dans l'étude les CGR jugés valides après les résultats des analyses sérologiques et prêts à être distribués au profit des patients. N'étaient pas inclus dans l'étude les CGR périmés, les CGR dont la poche est fissurée, les CGR contenant des caillots et enfin les CGR hémolysés.

**Taille de l'échantillon et plan d'échantillonnage:** nous avons employé une méthode non destructrice pour le choix des poches de CGR incluses dans notre étude. Les poches de CGR étaient choisies, tous les jours de la semaine, au hasard dans le lot des poches qui étaient prêtes à entrer en stock pour la distribution. Pour une meilleure analyse statistique, nous avons souhaité inclure plus de 1% de la production trimestrielle de CGR au CNTS de Lomé. Ainsi, nous avons analysé 260 poches (dont 204 unités adultes et 56 unités pédiatriques) représentant environ 3,2% de la production trimestrielle de CGR. La détermination des paramètres hématologiques se réalisait au fur et à mesure que les PSL étaient sélectionnés. Les résultats obtenus étaient notés dans un fichier Excel.

**Variables qui étaient étudiées:** pour chaque CGR, nous avons déterminé le volume de la poche. L'hématocrite et le contenu en hémoglobine ont été déterminés grâce à l'automate dont nous avons évalué la fidélité et la justesse afin d'assurer la qualité de nos mesures selon la recommandation de la norme ISO 15189 version 2012. Nous n'avons pas évalué le taux d'hémolyse à la fin de la conservation car il y avait un problème de disponibilité des CGR. Nous avons travaillé sur les CGR frais car plus de 95% des CGR produits étaient immédiatement distribués. De même, nous n'avons pas déterminé le taux de leucocytes résiduels car les CGR n'étaient pas des leucocytes avant leur transfusion.

**Volume de CGR contenu dans chaque poche:** nous avons pesé les poches de CGR à l'aide de la balance numérique qui était étalonnée. Nous avons également pesé à l'aide de la même balance la masse d'une poche vide. Les valeurs ont été consignées dans le fichier Excel. Le volume de CGR contenu dans chaque poche a été obtenu selon la formule ci-après: Volume du CGR= (Masse totale du CGR-Masse de la poche vide)/(Densité du CGR). **NB:** masse volumique du CGR = 1.06g/ml.

**Quantité d'hémoglobine et hématocrite des poches de CGR:** par stripping, on homogénéise le contenu de la tubulure et le contenu de la poche de CGR. Grâce à une scelleuse, on subdivise la tubulure en petits segments d'environ 6cm chacun appelé «boudi». On a détaché les deux derniers boudins et on a transféré leur contenu dans un tube EDTA qui était conservé entre 2 et 6°C. Ensuite, on a réalisé l'hémogramme sur l'aliquote du tube EDTA. Le contenu en hémoglobine de chaque poche de CGR analysée était calculé à partir de la formule suivante: Quantité Hb (en g)= taux d'Hb (en g/dl) x volume de la poche (en dl).

**Sexe et âge des donneurs et antécédent de dons en 2017:** nous avons trouvé les informations concernant les donneurs notamment le sexe, l'âge et les antécédents de dons en 2017 grâce à «TransfusControl^®^» qui était le logiciel de gestion des données des activités transfusionnelles du CNTS de Lomé.

**Comparaison des données aux normes internationales:** les données obtenues ont été comparées aux critères qualités des CGR décrits dans le guide européen [6]. Il s'agit notamment du volume, de la quantité d'hémoglobine et de l'hématocrite. D'après le guide européen, le CGR unité adulte (CGRUA) doit avoir un volume= 280±50ml, contenir au moins 45g d'Hb et avoir un hématocrite compris entre 65 à 75%. Pour le CGR unité pédiatrique (CGRUP), le volume minimal est 125ml, la quantité d'Hb doit être supérieure à 25g et l'hématocrite compris entre à 65 à 75%.

**Saisie et analyse des données:** les résultats obtenus ont été analysés grâce aux logiciels R studio 3.3.2. Le test «t» de *student* et le test de Fisher exact ont été utilisés. La valeur p<0,05 a été retenue comme seuil statistiquement significatif.

## Résultats

**Caractéristiques des donneurs dont les CGR ont été analysés:** les CGR de notre étude provenaient essentiellement des donneurs de sexe masculin: 92,16% (n=242) contre 7,84% (n=18) obtenus à partir du sang prélevé chez les femmes. L'âge moyen des donneurs était de 28,72±7,03 ans avec des extrêmes allant de 18 à 54 ans. La majorité des donneurs soit 92,70% (n=241) avaient un âge compris entre 18 et 39 ans.

**Volume des concentrés de globules rouges (CGR):** concernant les CGRUA, le volume moyen était 280,33±32,10ml avec des extrêmes allant de 219,5 à 386,6ml. Douze CGRUA (5,88%) avaient un volume inférieur à 230ml et 14,22% (n=29) avaient un volume supérieur à 330ml. Pour les CGRUP, leur volume moyen était 149,72±13,24ml avec des extrêmes allant de 123,7 à 189,3ml.

**Quantité d'hémoglobine des CGR:** pour les CGRUA, la quantité d'hémoglobine (Hb) moyenne était 54,82±10,64g avec des valeurs extrêmes respectives de 22,90g et 81,19g. Parmi les CGRUA, 18,14% (n=37) avaient une quantité d'Hb inférieure à 45g. Concernant les CGRUP, la quantité moyenne d'Hb était 28,28±6,45g avec des valeurs extrêmes de 14,83g et 40,14g. Dix-sept (30,36%) avaient une quantité d'Hb inférieure à 25g.

**Hématocrite des CGR:** l'hématocrite moyen des CGRUA était 62,11%±10,19 avec des valeurs extrêmes de 46,30% et 76,90%. Parmi les CGRUA, 43,13% (n=88) avaient un hématocrite conforme c'est-à-dire compris entre 65% et 75%. Au niveau des CGRUP, l'hématocrite moyen était 60,27±10,77% avec des extrêmes allant de 47,40 à 72,80%. Au total, sur les 260 CGR analysés 106 soit 40,77% étaient conformes pour tout l'ensemble des trois paramètres analysés à savoir le volume, le contenu en hémoglobine et l'hématocrite. Parmi les CGRUA, 42,16% (n=86) étaient conformes pour tous les paramètres contre 35,71% (n=20) pour les CGRUP. Les taux de conformités en fonction des différents paramètres sont indiqués dans le Tableau 1.

**Tableau 1:** taux de conformité des CGR en fonction des différents paramètres contrôlés

	Moyenne ± S	Normes européennes***	Pourcentage d’unités conformes (%)
Volume des CGR UA (ml)	280,33 ± 32,1	280 ± 50	79,90
Volume des CGR UP (ml)	149,72 ± 13,24	≥ 125	98,21
Quantité d’hémoglobine des CGR UA (g)	54,82 ± 10,64	≥45	81,86
Quantité d’hémoglobine des CGR UP (g)	28,28 ± 6,45	≥25	69,64
Hématocrite des CGRUA (%)	62,11 ± 10,19	65 à 75	43,13
Hématocrite des CGRUP (%)	60,27 ± 10,77	65 à 75	37,50

*** : normes européennes sur les spécifications des concentrés érythrocytaires;

S= écart-type; ml= millilitre; g= gramme; CGRUA: concentrés de globules rouges unités adulte; CGRUP: concentrés de globules rouges unités pédiatriques

**Conformité de la quantité d'hémoglobine selon le nombre de dons effectués en 2017:** les antécédents des donneurs de sang en termes du nombre de dons effectués au cours de l'année précédente ont eu une influence sur la qualité des CGR analysés. La conformité selon la quantité d´hémoglobine des CGRUA en fonction des antécédents de dons en 2017 des donneurs est indiquée dans le Tableau 2. Dans le même contexte, la conformité des CGRUP en fonction des antécédents de dons en 2017 des donneurs est indiquée dans le Tableau 3.

**Tableau 2:** conformité selon la quantité d'hémoglobine des CGRUA en fonction des antécédents de dons en 2017 des donneurs

	N	CGR non conformes		CGR conformes		P value
		n	%	N	%	
Antécédent de dons dans les 12 derniers mois						<0,05 **
0	44	5	11,36	39	88,64	
1	27	2	7,41	25	92,59	
2	31	4	12,90	27	87,10	
3	39	5	12,82	34	87,18	
4	63	21	33,33	42	66,67	
**Total**	**204**	**37**	**18,14**	**167**	**81,86**	

** = Test exact de Fisher;

N= effectif des donneurs de sang; n= effectif des CGR UA

**Tableau 3:** conformité selon la quantité d'hémoglobine des CGRUP en fonction des antécédents de dons en 2017 des donneurs

	N	CGR non conformes		CGR conformes		P value
		n	%	N	%	
Antécédent de dons dans les 12 derniers mois						<0,05**
0	2	0	0	2	100	
1	7	0	0	7	100	
2	13	2	15,38	11	84,62	
3	12	4	33,33	8	66,67	
4	22	11	50	11	50	
**Total**	**56**	**17**	**30,36**	**39**	**69,64**	

** = Test exact de Fisher

N= effectif des donneurs de sang, n= effectif des CGR UP

## Discussion

Nous avons choisi le laboratoire d'hématologie de l'INH pour l'analyse de nos échantillons parce qu'il était accrédité ISO 15189 pour l'hémogramme. En plus, ce laboratoire avait effectué le mois avant l'analyse de nos échantillons une vérification sur site de la méthode selon les recommandations de la norme ISO 15189:2012 et du document SH GTA 04 de Cofrac [7].

Le volume moyen des CGRUA était 280,33±32,1ml. C'était similaire à ce qu'a rapporté Eiman *et al.* soit 275±35,5ml [8]. Sur les 204 CGRUA, 163 unités (79,90%) avaient un volume conforme aux normes tandis que 29 unités (14,22%) avaient un volume au-dessus des 330ml. Par contre Fétéké *et al.* [5] au Togo en 2006 avaient trouvé un taux de conformité de 21,48% et la majorité du reste (77,78%) avait un volume en dessous de la norme dans leur contrôle. La différence entre les deux résultats s'expliquerait par le fait que la recommandation faite par Fétéké *et al.* d'ajuster le volume de sang total prélevé chez le donneur avait été mise en œuvre en utilisant systématiquement une balance pèse-poche lors du prélèvement du sang chez les donneurs. Mbanya *et al.* [9] au Cameroun en 2007 avaient trouvé un taux de conformité de 57% en ce qui concerne le volume des CGR. Ce taux était en deçà de notre résultat et pourrait s'expliquer par le temps qui sépare leur étude de la nôtre. Cependant, l'unité de préparation des PSL du CNTS devra mieux ajuster le volume de plasma à extraire afin d'améliorer encore le taux de conformité pour ce paramètre.

Dans notre étude, 55 sur 56 CGRUP soit 98,21% étaient conformes concernant le volume mais, la tendance allait vers des CGRUP de grand volume: près de la moitié soit 46,43% (n=26) étaient supérieurs à 150ml. On pourrait penser à une extraction insuffisante du plasma lors de leur préparation. Ceci a eu une incidence sur l'hématocrite des unités pédiatriques dont seulement 37,50% étaient conformes aux valeurs normales. Il serait nécessaire de revoir le processus de préparation des unités pédiatriques afin d'extraire un volume adéquat de plasma sans impact ni sur le volume final du CGRUP ni sur l'hématocrite recommandés.

Concernant le contenu en hémoglobine, 81,86% (n=167) des unités adultes contrôlées étaient normales. Ce résultat est comparable à celui de Fétéké *et al.* [5] qui avaient trouvé un taux de conformité de 80,74%. Mbanya *et al.* [9] au Cameroun en 2007 avaient constaté que 66% des poches de CGR étaient conformes pour leur contenu en hémoglobine. Cependant il serait souhaitable d'inclure la détermination du taux d'hémoglobine dans la sélection médicale afin d'atteindre, pour le contenu en hémoglobine, un taux de conformité d'au moins 90% tel que le recommande le guide européen [6].

Contrairement aux CGRUA où 81,86% avaient une quantité d'hémoglobine conforme, seulement 69,64% des CGRUP étaient conformes pour ce paramètre. Il faudrait donc améliorer le processus de la sélection médicale des donneurs spécialement pour les poches pédiatriques. Déterminer le taux d'hémoglobine avant le prélèvement de sang destiné à faire des unités pédiatriques serait l'idéal pour éviter les donneurs anémiés d'une part et améliorer la qualité des unités pédiatriques d'autre part [2]. Les moyennes de l'hématocrite étaient 62,11±10,19 et 60,27±10,77 respectivement pour les CGRUA et CGRUE. Au Pakistan, Sultan S *et al.* ont retrouvé un résultat similaire soit 69.5±7.24 sur 400 CGR contrôlés [3]. Pour l'hématocrite, 43,13% (n=64) des CGRUA étaient conformes. Il y a donc une amélioration significative de ce paramètre car Fétéké *et al.* [5] avaient constaté en 2006, que 20% des unités étaient conformes aux normes en matière d'hématocrite. Au Cameroun, Mbanya *et al.* [9] avaient un taux de 95% d'unité conforme par rapport à l'hématocrite. Ces différences seraient dues aux valeurs de références considérées par les différentes équipes.

Dans notre étude, une partie des poches non conformes pour l'hématocrite serait due à une insuffisance du volume de plasma qui était extrait après la centrifugation du sang total. En effet, nous avons trouvé que 14,22% des unités adultes avaient un volume au-dessus de 330ml et près de la moitié des CGRUP avaient un volume >150ml. Par conséquent, ajuster le volume de plasma à prélever lors de l'extraction corrigerait et le volume et l'hématocrite des CGR produits au CNTS de Lomé. Concernant la quantité d'hémoglobine, il y avait une différence significative (p=0,0126) entre les taux de conformité des CGRUA provenant des donneurs qui n'avaient pas fait de don ou qui avaient fait un seul don en 2017 comparés aux CGRUA provenant des donneurs ayant un antécédent de quatre dons en 2017. Le constat est le même au niveau des poches pédiatriques signifiant ainsi qu'il serait mieux d'éviter de faire plus que 4 dons dans l'année. La détermination du taux d'hémoglobine avant le prélèvement du sang chez les donneurs permettra un ajournement temporaire des donneurs anémiés pour respecter les règles de la sélection médicale des donneurs de sang [2, 6, 7].

Le pourcentage de poches conformes concernant les trois paramètres (volume, quantité d'hémoglobine et hématocrite) était 42,16% (n=86) pour les unités adultes et 35,71% pour les unités pédiatriques. Fétéké *et al.* [5] avaient trouvé en 2006, dans le même établissement de transfusion sanguine, moins de 20% de CGRUA conformes. Il y a eu une amélioration du taux de conformité des CGR produits au CNTS de Lomé. Mais beaucoup d'efforts restent à faire pour atteindre le taux de conformité d'au moins 90% recommandé par le guide européen pour la préparation, l'utilisation et l'assurance de la qualité des composants sanguins [6]. Yao *et al.* [10] en Côte d'Ivoire en 2014 avaient trouvé que 93,85% des unités adultes et 98,71% des unités pédiatriques étaient conformes. Le résultat de Yao KD *et al.* était le fruit d'une expérience sur trois ans de la mise en place d'un laboratoire de contrôle qualité des produits sanguins labiles. Saloni *et al.* avaient aussi soutenu que le contrôle périodique de la qualité des produits sanguins était indispensable pour vérifier l'adéquation et la sécurité de ces produits et font partie des bonnes pratiques transfusionnelles [11]. Le CNTS de Lomé a donc un intérêt à mettre en place un laboratoire de contrôle qualité des produits sanguins labiles et améliorer les processus de production des CGR si le centre veut fournir des CGR de meilleure qualité aux patients.

## Conclusion

Globalement 42,16% et 35,71% respectivement des unités adultes et des unités pédiatriques de CGR étaient conformes au complet. Ces résultats ont été obtenus dans des conditions techniques satisfaisantes puis qu'il y avait une évaluation de la justesse et de la fidélité des résultats rendus par l'automate. Il y a eu beaucoup d'amélioration par rapport au contrôle qui était réalisé en 2006. Cependant, beaucoup d'efforts restent encore à faire par le CNTS de Lomé pour atteindre le taux de conformité globale d'au moins 90% recommandé par le guide européen. Pour ce faire, nous suggérons que les actions correctives suivantes soient mises en œuvre: Ajuster le volume de plasma à extraire après la centrifugation du sang total, inclure le taux d'hémoglobine pré-don dans les critères d'aptitude au don de sang, élargir d'avantage l'intervalle entre deux dons de sang successifs, mettre en œuvre les activités du laboratoire de contrôle qualité qui fera régulièrement un contrôle de conformité des PSL en général et des CGR en particulier. Ces contrôles seront assortis de recommandations permettant de corriger en temps réel les éventuelles déviations dans la mise en application des procédures de production.

### Etat des connaissances actuelles sur le sujet

- L'existence des normes européennes définissant la qualité des concentrés de globules rouges. Ces normes devraient permettre d'assurer un standard dans la production de ce type de produit;
- L'impact du don de sang régulier sur le taux d'hémoglobine des donneurs;
- Très peu d'études africaines ont été publiées sur la qualité des produits sanguins labiles distribués. Elles montrent souvent un taux de conformité en dessous de 90% indiquant qu'il reste beaucoup d'effort à faire dans ce domaine.

### Contribution de notre étude à la connaissance

- L'existence des normes européennes définissant la qualité des concentrés de globules rouges. Ces normes devraient permettre d'assurer un standard dans la production de ce type de produit;
- L'impact du don de sang régulier sur le taux d'hémoglobine des donneurs;
- Très peu d'études africaines ont été publiées sur la qualité des produits sanguins labiles distribués. Elles montrent souvent un taux de conformité en dessous de 90% indiquant qu'il reste beaucoup d'effort à faire dans ce domaine.

## Conflits d’intérêts

Les auteurs ne déclarent aucun conflit d'intérêts.
